# Stress-Induced Takotsubo Cardiomyopathy After Transjugular Intrahepatic Portosystemic Shunt

**DOI:** 10.14309/crj.0000000000000377

**Published:** 2020-05-05

**Authors:** Bernadette Lamb, Benjamin Arbeiter, Neil Bhogal, John Anderson, Loretta Jophlin

**Affiliations:** 1 Department of Internal Medicine, University of Nebraska Medical Center, Omaha, NE; 2 Department of Gastroenterology and Hepatology, University of Nebraska Medical Center, Omaha, NE; 3 Department of Cardiology, University of Nebraska Medical Center, Omaha, NE

## Abstract

This report presents the first known documented case of Takotsubo cardiomyopathy, characterized by transient left ventricular systolic dysfunction after a transjugular intrahepatic portosystemic shunt procedure. A 59-year-old woman with decompensated cirrhosis underwent transjugular intrahepatic portosystemic shunt procedure with subsequent new-onset heart failure without previous diagnostic evidence of underlying cardiovascular disease, including cirrhotic cardiomyopathy. After 2 weeks of medical management with β-blockade and diuretics, the patient had recovery of a left ventricular ejection fraction.

## INTRODUCTION

Stress-induced (Takotsubo) cardiomyopathy is characterized by transient left ventricular systolic dysfunction in the absence of significant coronary artery disease or acute atherosclerotic plaque rupture.^1,2^ It is associated with both physical and emotional distress, from events such as surgical procedures, chemotherapy, asthma exacerbation, grief, relationship conflicts, and acute liver failure, and has been found to disproportionately affect postmenopausal women.^1–4^ We present the first known documented case of Takotsubo cardiomyopathy after a transjugular intrahepatic portosystemic shunt (TIPS) procedure.

## CASE REPORT

A 59-year-old woman with a medical history of decompensated cirrhosis secondary to primary biliary cholangitis underwent an elective TIPS procedure for refractory ascites. Three months before TIPS placement, a cardiac workup revealed a normal electrocardiogram. A 2-dimensional contrast-enhanced transthoracic echocardiogram (TTE) showed a left ventricular ejection fraction (LVEF) of 55%–60%. A dobutamine stress echocardiogram did not reveal evidence of inducible ischemia or systolic dysfunction.

A TIPS was placed, resulting in improvement of her hepatic venous pressure gradient from 8 to 4 mm Hg. Her ascites did not reaccumulate in the first 4 days after the procedure. However, she developed progressive respiratory distress with pulmonary edema visualized on thoracic radiograph. Cardiac evaluation performed 5 days post-TIPS placement with TTE demonstrated a newly reduced LVEF of 30%–35% and severe hypokinesis of all mid to distal segments, including the apex, along with hypercontractility of the basal segments. Electrocardiogram revealed new ST- and T-wave abnormalities in the anterolateral leads. Cardiac enzymes were not obtained. Coronary angiography revealed no significant epicardial coronary artery disease. Her new-onset cardiomyopathy was medically managed with β-blockade and diuretics. TTE 2 weeks after TIPS revealed improvement in her LVEF to 50%–55% with persistent hypokinesis of the apex and apical myocardial segments. TTE 2 months later revealed LVEF of 50%–55% and resolution of regional wall motion abnormalities. Unfortunately, the patient experienced progressively worsening hepatic encephalopathy after her TIPS procedure and required TIPS reversal. She ultimately underwent orthotopic liver transplantation.

## DISCUSSION

The TIPS procedure uses radiographic guidance to place a metal stent connecting the portal vein to the hepatic vein.^5,6^ This procedure is performed in cirrhotic patients with portal hypertension to reduce hepatic venous pressure gradient, therefore reducing the risk of bleeding from esophageal and gastric varices and ascitic fluid accumulation.^5,6^ TIPS is a nonsurgical intervention with relatively low risks. Previously reported complications of TIPS include infection, bleeding, hepatic encephalopathy, right-sided heart failure, and further decompensation of liver disease.^6^

Takotsubo cardiomyopathy is typically characterized by transient weakening of the left ventricle after a physical or emotional stressor in the absence of angiographic evidence of significant coronary artery disease or acute plaque rupture.^1,2^ Right ventricular involvement has been observed in stress-induced cardiomyopathy. However, its prognostic implications were mixed in the reported case series.^7–9^ Cases of isolated reversible right ventricular cardiomyopathy have also been reported.^10–12^ Interestingly, stress-induced cardiomyopathy has been found to disproportionately affect postmenopausal women.^2,3^ In the apical type of stress cardiomyopathy, similar to our patient, TTE reveals depressed function of mid and apical segments of the left ventricle and hyperkinesis of the basal segments.^13^ The regional wall abnormalities appreciated in stress-induced cardiomyopathy extend beyond a singular vascular territory, differentiating it from ischemic heart failure.^14^ The pathophysiology is not well understood. However, it has been postulated that the cause may be related to catecholamine surge, microvascular dysfunction, or coronary artery spasm.^1,2^ Presenting symptoms may include acute-onset substernal chest pain, dyspnea, syncope, arrhythmias, or cardiac arrest.^2,3^ Individuals may have ST- or T-wave changes seen on electrocardiogram and mild elevations in cardiac enzymes.^2^ Systolic dysfunction generally improves or completely resolves within weeks to months.^1,2^ Takotsubo cardiomyopathy is a well-documented complication of postreperfusion syndrome after orthotopic liver transplant, although there are no documented cases of Takotsubo cardiomyopathy after a TIPS procedure.^15^

Cirrhotic cardiomyopathy (CC) is another etiology of heart failure in cirrhotic patients who undergo the TIPS procedure.^15^ CC is characterized by impaired systolic response to physical stress, diastolic dysfunction, and electrophysiological abnormalities (QT interval prolongation) in the absence of underlying cardiac disease.^16^ In some cases, CC is subclinical with mild systolic dysfunction noted on echocardiogram. Clinically significant disease may only manifest after a significant stressor, similar to the presentation of Takotsubo cardiomyopathy.^15^ In this case, the pre-TIPS TTE and dobutamine stress echocardiogram showed grade 2 diastolic dysfunction without left ventricular systolic dysfunction at rest or under stress. The lack of stress-induced systolic dysfunction before TIPS makes CC less likely compared with Takotsubo cardiomyopathy. Post-TIPS TTE showed primarily systolic dysfunction with minimal diastolic changes further supporting Takotsubo cardiomyopathy.

Our patient had a typical presentation of Takotsubo cardiomyopathy with reversible heart failure in the absence of coronary artery disease after a physical stressor, in this case, a TIPS procedure. Within days after TIPS, TTE revealed reduced ejection fraction and apical dyskinesis, which improved 2 weeks later and entirely resolved within a few months. This is the first known documented case of stress-induced cardiomyopathy as a complication of the TIPS procedure.

## DISCLOSURES

Author contributions: All authors contributed equally to this manuscript. L. Jophlin is the article guarantor.

Financial disclosure: None to report.

Informed consent was obtained for this case report.
